# Comparison of Nutritional Components, Ruminal Degradation Characteristics and Feed Value from Different Cultivars of Alfalfa Hay

**DOI:** 10.3390/ani13040734

**Published:** 2023-02-18

**Authors:** Xinyue Zhang, Yanfang Liu, Fanlin Kong, Wei Wang, Shengli Li

**Affiliations:** 1 State Key Laboratory of Animal Nutrition, Beijing Engineering Technology Research Center of Raw Milk Quality and Safety Control, College of Animal Science and Technology, China Agricultural University, Beijing 100193, China; 2 Beijing Sino Agricultural Aiko Testing Technology Co., Ltd., Beijing 100193, China

**Keywords:** alfalfa hay, nutrient contents, rumen degradation, feed value

## Abstract

**Simple Summary:**

The nutrient contents and rumen degradation of alfalfa hay rely highly on cultivars and grown regions. Moreover, the evaluation of alfalfa hay with respect to nutritional components in conjunction with the selection of feed resources according to local condition is necessary and essential for dairy farms to obtain an efficient and economic formula. Thus, this study was conducted to investigate the variations of different alfalfa hay cultivars grown in China and the US in terms of nutritional components, ruminal degradation characteristics and feed value. The results showed that the cultivar of American Golden Empress (GE) had the greatest rumen degradation characteristics; meanwhile, the indicators of total digestible nutrients (TDN), dry matter intake (DMI), digestible dry matter (DDM), relative feed value (RFV) and relative forage quality (RFQ) of GE were better than China Zhongmu No. 1 (ZM1) and China Gongnong No. 1 (GN1) cultivars.

**Abstract:**

The objective of this study was to evaluate the effects of different cultivars of alfalfa hay, including American Anderson (AA), American Golden Empress (GE), China Zhongmu No. 1 (ZM1) and China Gongnong No. 1 (GN1), on conventional nutrient composition, rumen degradation characteristics and feed value. Four healthy Holstein cows (137 ± 14 days in milk, 2.40 ± 0.50 parity) equipped with permanent ruminal cannulas were examined for the nylon-bag technique. The alfalfa hay samples were incubated in the rumen for 0, 4, 8, 12, 24, 36, 48 and 72 h according to the “gradual in/all out” schedule to detect the ruminal nutrients’ degradability. Our results showed that various cultivars of alfalfa hay from different planting regions had significant differences on nutrient contents, rumen degradability and feed value. For nutritional components of alfalfa hay, the highest dry matter (DM) content was found in GE and the lowest in GN1 (*p* < 0.001); however, GN1 had the greatest concentration of ether extract (EE, *p* = 0.01), Ca (*p* < 0.001) and the lowest Ash (*p* < 0.001). Additionally, the lowest neutral detergent fiber (NDF), acid detergent fiber (ADF) and highest starch contents were observed in AA and GE (*p* < 0.001). Meanwhile, the cultivar of ZM1 represented the highest NDF, ADF and Ash contents, in conjunction with minimal CP and P concentrations (*p* < 0.001). In terms of rumen degradation characteristics, the effective degradation rate (ED) of DM in GE and ZM1 was significantly higher than that in AA and GN1 (*p* = 0.013). The NDF effective degradation was lower in ZM1 than the other three cultivars (*p* = 0.002), and in addition ZM1 also showed lower CP and ADF effective degradation than GE (*p* < 0.001). As far as feed value was concerned, the cultivar of alfalfa hay imported from the US, including AA and GE, exhibited higher relative feed value (RFV) and relative forage quality (RFQ) than Chinese alfalfa based on ZM1 and GN1 (*p* < 0.001). In conclusion, the results suggested that the cultivar of GE exhibited greater rumen degradable characteristics and feed value, while ZM1 showed the opposite status.

## 1. Introduction

As a major source of fiber, forages are especially important for ruminant animals such as cows to stay healthy. Firstly, the cultivar of roughage has a huge impact on the milk performance of dairy cows. Wang et al. reported that cereal straw replaced alfalfa as the main forage source contributing to lower milk protein yield [1]. Then, the daily performance of dairy cows could change according to the length or storage method of forages. Haselmann et al. pointed out that the reduction in the particle size of forages increased cows’ time budget for lying, and the preservation approach of forage (silage vs. hay) modulated the laterality of lying behavior [2]. In addition, the relationship between the quantity of roughage intake by ruminants and energy utilization (or greenhouse gas emission) was also investigated [3]. More importantly, as forage stock shortages increased, frequently the operating cost comprised by feedstuffs grown steadily [4]. Thus, evaluating roughages based on nutrient contents and further selecting the best resources according to local condition are essential for dairy farms to obtain an efficient and economic formula.

As the “king of forage”, alfalfa has attracted plentiful attention to its higher concentration of soluble saccharides and more easily available protein [5,6]. Currently, alfalfa, a perennial legume crop, is considered as the most common and commercial feedstuff utilized for dairy rations in many countries [7,8]. Dairy cows fed alfalfa-based diets exhibited better production performance compared to corn stover or Chinese wild rye grass (*Leymus chinensis*) hay [9]. However, due to the north–south gap regarding temperature, soil and precipitation in China, uneven nutritional components were identified [10]. On the other hand, the expansion of dairy farms contributed to the shortage of feedstuff resources with the vigorous development of animal husbandry. Nowadays, Chinese high-quality roughages mainly rely on imports. In 2021, a total of 1.78 million tons of alfalfa hay was imported, and the US accounted for 80.6% [11]. Therefore, it is necessary and essential to estimate and point out the disparity in alfalfa hay between China and the US.

In 2018, the China Animal Husbandry Association promulgated a standard of “Alfalfa Hay Quality Classification”, and a number of studies related to grade evaluation were implemented [12,13], but there was unexplored information on the comparison of nutrient contents and rumen degradation in Chinese and American alfalfa, although the assessment was applicable to the different countries’ alfalfa cultivars. Above all, the objective of this study was to analyze the difference in the nutritional components, rumen degradable characteristics and feed value of different alfalfa hay cultivars from China and the US to supply the basal data for the dietary formulation of dairy farms, in conjunction with providing a reference for replacing a portion of alfalfa hay for dairy cows.

## 2. Materials and Methods

### 2.1. Alfalfa Cultivars and Grown Regions

Four cultivars of alfalfa hay grown in China and the US were selected for this study, including China Zhongmu No. 1 (ZM1), China Gongnong No. 1 (GN1), American Anderson (AA) and American Golden Empress (GE). Specific information related to the experimental samples is summarized in Table 1. Twenty plants from each plot were randomly harvested, and a height of 10 cm above the ground was selected. The exterior 1 m area of each plot was excluded from sampling to ensure uniformity in the sampled plants. Then, these samples were sent to the laboratory in clean plastic bags for further analysis.

### 2.2. Nutritional Components and Feed Value

The samples of alfalfa hay were dried in a forced oven set at 65 °C for 48 h, weighed to record dry matter (DM) content, and then ground through a 1.0 mm screen for chemical analysis or through a 2.5 mm screen for rumen degradation characteristics detection. The concentrations of DM (method 950.15), crude protein (CP, method 2001.11), ether extract (EE, method 920.39), ash (method 942.05), starch (method 996.11), calcium (Ca, method 965.09) and phosphorus (P, method 965.17) were measured according to AOAC (2016) [14]. The NDF and ADF contents were analyzed using an Ankom Fiber Analyzer System (Ankom Technology, Macedon, NY, USA) as Van Soest [15] mentioned.

The feed value based on total digestible nutrients (TDN), dry matter intake (DMI), digestible dry matter (DDM), relative feed value (RFV), and relative forage quality (RFQ) were estimated as follows:

(1) TDN%=82.38−(0.7515×ADF)

(2) DMI%=120∕NDF

(3) DDM%=88.9−0.779×ADF

(4) RFQ%=DMI×TDN/1.23

(5) RFV%=DMI×DDM/1.29

### 2.3. In Situ Rumen Degradation

A total of four healthy lactating (137 ± 14 days in milk, 2.40 ± 0.50 parity) Holstein dairy cows fixed with a permanent rumen fistula from ZhongDi Dairy Holdings Company Limited (40°11′ N 116°88′ E, Beijing, China) were allocated as the donors of rumen fluid in this study. The basal diet was formulated as TMR according to recommendations in the NRC (2001) guidelines [6]. The donor animals were fed twice daily (07:30 and 18:30) for ad libitum intake, aiming for 3 to 5 kg residues. Forty percent of the daily feed allowance was supplied in the morning and sixty percent in the afternoon. The ingredients and nutrient compositions of the basal diet are listed in Table 2.

Each alfalfa hay sample (ca. 4 g) was randomly incubated in sealed nylon bags (8 cm × 12 cm, pore size 40–60 μm), and two replicates per cultivar from individual cows were settled at each incubation time point. Every eight sampled bags were bounded to the end of a soft polyethylene plastic tube (around 50 cm), which then was inserted into the abdominal sac of rumen. Additionally, the other side of the polyethylene plastic tube was tied with a strong nylon rope connected a little iron ring, which was exposed outside of the fistula plug to prevent the nylon bags from falling into the rumen. A total of eight tubes were placed in the rumen of each experimental cow for 0, 4, 8, 12, 24, 36, 48 and 72 h, respectively, according to the “gradual in/all out” schedule. After incubation, all nylon bags were taken out of the rumen and washed with cold running tap water until the outlet water was clear. Then, all washed nylon bags were dried in the forced air oven at 65 °C for 48 h. The dried residues of two replicate nylon bags per sample at same time point from individual cows were mixed and stored in plastic sealed bags for further analysis. The rumen degradation kinetic parameters were calculated as follows:

Degradation rate of alfalfa hay at different time points:

(6) A(%)=[(B−C)/B]×100%

where A is the rumen degradation rate of the nutritional component of a sample at a specific time; B is the nutritional content of the sample; and C is the residue of the sample in the nylon bag.

Rumen degradation kinetic parameters and effective degradation rate were estimated according to the method explained by Ørskov and McDonald [16], and the equation was described as:

(7) $$P=a+b(1-e^{-\mathrm{ct}})$$

where P stands for the rumen degradation rate of the nutritional component of a sample at a specific time; a represents the rapid degradation fraction; b is the tslow degradation fraction; c stands for the degradable rate constant of slow degradation fraction; and t equals lag time in rumen.

(8) ED (%)=a+bc/(k+c)

where ED is the effective degradation rate, and k stands for the estimated rate of outflow from the rumen (h^−1^) and is assumed to be 0.031 h^−1^ in this study.

### 2.4. Statistical Analysis

Data on nutritional components and rumen degradation kinetic parameters were established by one-way ANOVA using the model of non-linear procedure in SAS 9.2 software (SAS institute, Carry, NC, USA), and the Duncan method was used to analyze the multiple comparison based on the following model:

(9) $$Y_{\mathrm{ijk}}=\mu +T_{i}+D_{j}+e_{\mathrm{ijk}}$$

where Y_ijk_ represent the nutritional components, real-time degradable rate and degradable related parameters, μ stands for the overall average, T_i_ means different cultivars of alfalfa hay, D_k_ is the random effect of dairy cow, and e_ijk_ is the model error.

For all statistical analyses significant difference was declared at *p* < 0.05, whereas tendency was identified as 0.05 ≤ *p* ≤ 0.1.

## 3. Results

### 3.1. Difference in Nutritional Components of Various Cultivars of Alfalfa Hay

The nutritional profiles of four cultivars of alfalfa hay were represented in Table 3. The concentration of DM varied from 89.86% to 92.10%, and the cultivar of GE and GN1 showed the highest and lowest content, respectively (*p* < 0.01). The AA cultivar had greater CP content (19.02%) than the GN1 (18.41%), GE (18.29%) and ZM1 (16.05%) groups (*p* < 0.01). The lowest concentrations of NDF and ADF were observed in AA and GE, but the highest value was detected in the ZM1 (*p* < 0.01) cultivar. As for the concentration of EE, GN1 (2.57%) was higher than the other three counterparts (*p =* 0.01). The greatest ash content was recorded for ZM1 (9.00%), followed by GE (8.73%) and AA (8.52%), whereas the lowest ash content occurred in GN1 (7.91%, *p* < 0.01). In addition, the starch content was significant higher in AA (4.25%) and GE (4.10%) than that in ZM1 (3.91%) and GN1 (3.78%, *p* < 0.01), but no difference was found in AA and GE. In terms of minerals, P content varied among different cultivars, with the highest content estimated for GE (0.36%) and lowest for ZM1 (0.23%, *p* < 0.01); however, the concentration of Ca presented the same result in GE (1.24%) and ZM1 (1.24%), which was marked lower than that in AA (1.34%) and GN1 (1.34%, *p* < 0.01).

### 3.2. Difference in CP Rumen Degradation of Various Cultivars of Alfalfa Hay

**Figure 1:**
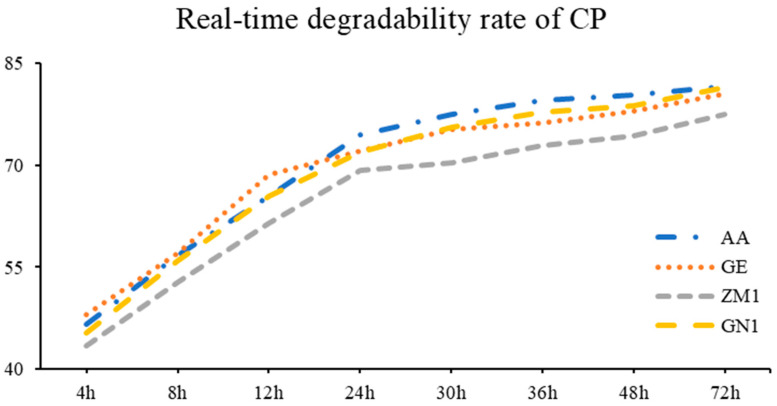
The real-time degradability rate of CP in different alfalfa hay cultivars. AA: American Anderson, GE: American Golden Empress, ZM1: Zhongmu No. 1, GN1: Gongnong No. 1.

The rumen real-time degradability rate and degradable characteristics are summarized in Table 4. ZM1 showed the lowest CP degradation rate when it stayed in the rumen for 12 h, 30 h, 36 h, 48 h and 72 h (*p* < 0.05; Figure 1), especially at the time point of 30 h and 72 h, where the degradation rates of ZM1 were 70.38% and 77.48%, respectively. The other three cultivars varied from 75.32% to 77.47%, as well as 80.58% to 81.61%, respectively (*p* < 0.01). In terms of CP degradable characteristics, a, b and c were not affected by the cultivar of alfalfa hay (*p* > 0.05). However, the highest values of a and b were found in AA (81.40%) and GN1 (80.37%), which were followed by GE (78.54%) and ZM1 (75.68%, *p* < 0.01). The cultivars of AA (68.50%) and GE (68.28%) had greater ED of CP than ZM1 (63.69%) and GN1 (67.30%, *p* < 0.01).

### 3.3. Difference in DM Rumen Degradation of Various Cultivars of Alfalfa Hay

**Figure 2:**
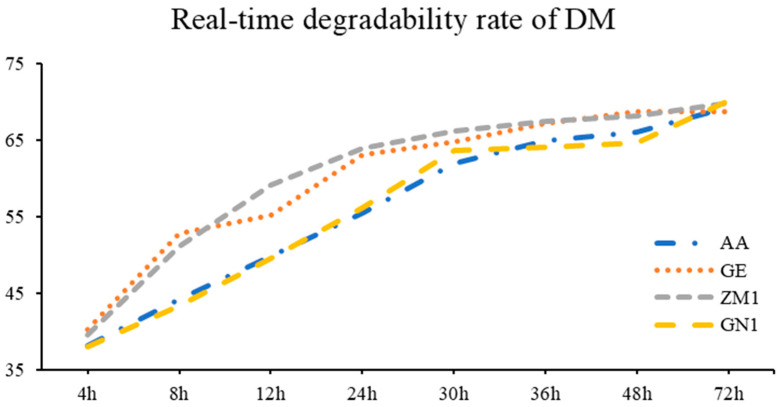
The real-time degradability rate of DM in different alfalfa hay cultivars. AA: American Anderson, GE: American Golden Empress, ZM1: Zhongmu No. 1, GN1: Gongnong No. 1.

As shown in Table 5 and Figure 2, the DM degradability of GE and ZM1 were far higher than AA and GN1 at the time points of 8, 12, 30, 36 and 48 h (*p* < 0.05). As for degradation parameters, the alfalfa hay cultivars of AA and GN1 showed greater c and ED than GE and ZM1 (*p* = 0.01), but the degradation parameters of a and b were not influenced by different alfalfa hay cultivars.

### 3.4. Difference in NDF Rumen Degradation of Various Cultivars of Alfalfa Hay

**Figure 3:**
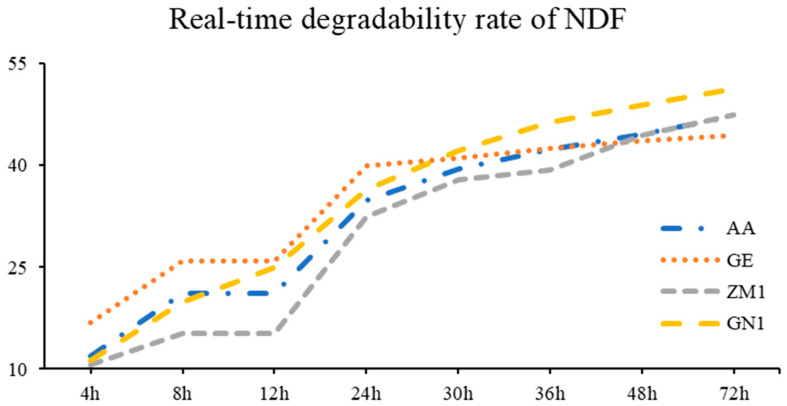
The real-time degradability rate of NDF in different alfalfa hay cultivars. AA: American Anderson, GE: American Golden Empress, ZM1: Zhongmu No. 1, GN1: Gongnong No. 1.

Table 6 and Figure 3 suggested that the NDF rumen degradation of the GE cultivar was significantly higher than that in AA, ZM1, and GN1 at 4 h, 8 h and 24 h (*p* < 0.05), whereas GN1 took the lead in NDF rumen degradation since the lag time of 36 h (*p* = 0.02) was achieved. For degradable indicators, a was estimated greater in GE (6.89%) compared to AA (2.73%), ZM1 (2.39%) and GN1 (2.05%, *p* = 0.03), but GE exhibited lower b than others (*p* < 0.01; GE: 37.94% < AA: 43.51% < ZM1: 47.66% < GN1: 51.11%). Additionally, the lowest ED was found in ZM1 (30.06%, *p* = 0.02); meanwhile, no disparity was represented among the other three cultivars of alfalfa hay (AA, GE, GN1).

### 3.5. Difference in ADF Rumen Degradation of Various Cultivars of Alfalfa Hay

**Figure 4:**
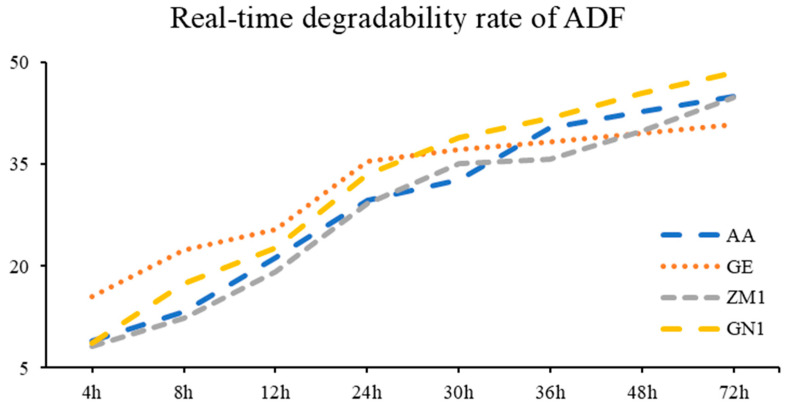
The real-time degradability rate of ADF in different alfalfa hay cultivars. AA: American Anderson, GE: American Golden Empress, ZM1: Zhongmu No. 1, GN1: Gongnong No. 1.

The real-time degradability rate and degradation parameters of different alfalfa hay cultivars were summarized in Table 7, in conjunction with the visualization, which was pictured in Figure 4. The highest ADF degradation after 4 h, 8 h and 12 h in the rumen was estimated in GE, compared to AA, ZM1 and GN1 cultivars (*p* < 0.01). Both GE and GN1 showed the greatest degradation of ADF until the lag time in rumen up to 24 h and 30 h (*p* < 0.05). At the time point of 48 h and 72 h, GN1 was far higher than the other three cultivars of alfalfa hay (*p* = 0.01). For degradation parameters, a and a + b of ADF were greatest in GE (*p* < 0.01), and no significant difference was estimated among the other three cultivars. In addition, ED was marked higher in GE (30.73%) and GN1 (30.84%) compared to AA (28.23%) and ZM1 (27.01%, *p* < 0.01), but no difference was found between GE and GN1 or AA and ZM1.

### 3.6. Difference in Feed Value of Various Cultivars of Alfalfa Hay

The feed value evaluations of various cultivars of alfalfa hay are presented in Table 8. It was clearly shown that the greatest TDN, DMI, DDM, RFV and RFQ were estimated in AA and GE, which was followed by GN1, and the cultivar of ZM1 exhibited the lowest value in the above five indicators (*p* < 0.01).

## 4. Discussion

A high DM content in forage supplies more nutrients for ruminants and leads to an easier preservation method [17]. In the current study, the significantly high DM content in GE indicated that alfalfa planted in the US had greater available nutrients and a longer storage time. The result was consistent with a previous study, where Xiong et al. suggested that the US alfalfa hay had the highest DM content [18]. It is generally thought that high CP content represented high digestibility, and the N supplementation utilized by rumen microorganism also was improved [19]. In agreement with Zhang et al. [20], our study found that the cultivar of AA had the highest CP content. The concentrations of NDF and ADF are important parameters to estimate the degradability and utilization of roughages. In this study, lower NDF and ADF contents were observed in two cultivars of American alfalfa hay compared to other two Chinese alfalfa hay cultivars, which suggested that alfalfa hay grown in American was more easily digested and utilized. Additionally, the results were in line with Wang et al. [21], but different from Xiong et al. [18]. The inconsistencies among studies could be explained by various growing conditions (light, temperature, precipitation, soil status) or genetic characteristics [22,23,24]. It is reported that a higher soil pH is more easily accelerates the deposition of fatty acids in plants [25], which means that the higher EE content in GN1 may the result of soil salinity. The cultivar of AA from America exhibited a lower ash content compared to ZM1 from China, and such a result could be attributed to the smooth harvesting ground in America. In detail, more soil would be taken into the feedstuffs, and then higher ash content would be detected when the ground flatness was poor. Starch is one of the major factors to influence cows’ milking performance [26]. The results related to starch content in our research verified that the alfalfa hay grown in the US may have greater milking quality. Mineral contents were always ignored because of the addition of premixes in ruminant diets; however, their inclusion in roughages also represent a vital position. The highest Ca content in AA and GN1 and greatest P content in GE were observed in current study, and they were in accordance with Yang et al. [27].

It is reported that the more CP content in forage, the higher CP degradation in rumen; our result was consistent with the theory. Moreover, the ED of CP in alfalfa hay planted in the US was far higher than that in China, which was in agreement with Balgees et al. [28]. The difference in rumen CP degradation between Chinese and American cultivars could be explained by the effective area of rumen microbial invasion to feeds and protein structure [29,30]. Dry matter intake is largely affected by the DM rumen degradation [31]. The value of the DM degradation rate increased gradually with the growth lag time in rumen, and then tended to be stable, which was in line with Saleem et al. [32]. Additionally, the ED of DM in GE and ZM1 were 58.67% and 58.90%, respectively, which meet the criterion of first-grade alfalfa hay [33]. The degradation of NDF and ADF is the reflection of the nutritional utilization [34]. In the current study, the ED of NDF in alfalfa hay from two different regions was lower than that found by Eun et al. [35]. The disparity between different experiments may be due to the animals’ health status or the type of basal diet. Moreover, consistent with a previous study [33], the rumen degradation of ADF and NDF from all four cultivars of alfalfa hay mainly occurred within 48 h.

Feed value is a comprehensive index to assess the quality of feedstuffs [36]. TDN, the reflection of roughage digestibility and animal digestion, evaluates the nutritional value of forage based on total digestible nutrients [37]. Generally, roughage with high CP content and low NDF and ADF contents represent great TDN [38]. In the current study, the CP content of American alfalfa was higher, whereas the NDF and ADF contents were lower than that in alfalfa hay grown in China. The results indicated that the quality of alfalfa hay planted in the US was better based on the evaluation of TDN. As an important quality reference of alfalfa in the US, RFV is a comprehensive method based on the estimation of NDF and ADF [39]. However, the analysis of RFV fails to take CP content into account. RFQ is closer to the actual situation than RFV, and can grade forages precisely [40]. In agreement with the previous report of Liu et al. [41], our research also suggested that RFQ in alfalfa hay grown in the US was far higher than in Chinese hay. However, Xiong et al. [18] found opposite results; they thought that American alfalfa hay had lower TDN, RFV and RFQ. The different results may highly be influenced by various sample cultivars selected in these reports.

## 5. Conclusions

In conclusion, the cultivar of GE from the US had the greatest rumen degradation characteristics, and meanwhile the indicators of TDN, DMI, DDM, RFV and RFQ of GE were all better than ZM1 and GN1. These results can supply the basic data for the dietary formulation of dairy farms and help them seek other unconventional feedstuffs adapted to local conditions. Additionally, we would like to develop a rapid, efficient and economical analytical method for predicting the nutrient contents and rumen degradability of alfalfa hay based on various cultivars and grown regions.

## Figures and Tables

**Table 1:** Samples information.

Items	Sample Description	Growing Area	Sampling Time	Longitude	Latitude
AA	Anderson, budding stage	Wisconsin, USA	2016.09.18	86°49′~92°54′ W	42°30′~47°30′ N
GE	Golden empress, first flowering	Ohio, USA	2016.10.04	80°32′~84°49′ W	38°27′~41°58′ N
ZM1	Zhongmu No. 1, first flowering	Zhangjiakou, China	2016.10.04	113°50′~116°30′ E	39°30′~42°10′ N
GN1	Gongnong No. 1, first flowering	Fujin, China	2016.09.24	131°25′~133°26′ E	46°45′~47°45′ N

Note: AA: American Anderson, GE: American Golden Empress, ZM1: Zhongmu No. 1, GN1: Gongnong No. 1.

**Table 2:** Composition and nutrient levels of basal diet. (DM basis %).

Ingredients	Contents %
Chinese wildrye	6.38
Alfalfa hay	20.31
Oat hay	5.58
Wheat	1.76
Corn silage	24.48
Corn	3.66
Steam-flaked corn	13.52
Soybean meal	6.55
Extruded soybean	3.76
Soybean hull	3.00
Cottonseed	3.41
Molasses	3.80
Rumen-pass fatty acid	1.20
Yeast powder	0.20
Mycotoxin removement agent	0.06
NaCl	0.31
Limestone	0.32
Ca(HCO_3_)_2_	0.34
NaHPO_3_	0.67
KHCO_3_	0.26
Premix ^(1)^	0.31
MgO	0.12
NE_L_ (MJ/kg) ^(2)^	5.68
CP	15.01
EE	3.40
NDF	41.03
ADF	26.69
Ca	0.55
P	0.39

(1) One kg premix contained the following: VA, 1,000,000 IU; VD3, 280,000 IU; VE, 10,000 IU; nicotinic acid, 1000 mg; Cu (as copper sulfate), 3250 mg; Mn, 4800 mg; Zn, 12,850 mg; I, 140 mg; Se, 150 mg; Co, 110 mg; (2) NE_L_ is net energy of lactation, a calculated value according to NRC 2001 [8], while the other nutrient contents were measured values.

**Table 3:** The nutritional contents of various alfalfa hay cultivars (DM basis) %.

Items ^1^	DM	CP	NDF	ADF	EE	Ash	Starch	Ca	P
AA	91.11 ^c^	19.02 ^a^	43.76 ^c^	32.99 ^c^	2.30 ^bc^	8.52 ^c^	4.25 ^a^	1.34 ^a^	0.34 ^b^
GE	92.10 ^a^	18.29 ^b^	43.18 ^c^	34.02 ^c^	2.18 ^c^	8.73 ^b^	4.10 ^a^	1.24 ^b^	0.36 ^a^
ZM1	91.17 ^b^	16.05 ^c^	53.63 ^a^	42.30 ^a^	2.38 ^b^	9.00 ^a^	3.91 ^b^	1.24 ^b^	0.23 ^d^
GN1	89.86 ^d^	18.41 ^ab^	49.08 ^b^	38.69 ^b^	2.57 ^a^	7.91 ^d^	3.78 ^b^	1.34 ^a^	0.25 ^c^
SEM	0.014	0.205	0.331	0.361	0.065	0.063	0.052	0.011	0.002
*p*-value ^2^	<0.01	<0.01	<0.01	<0.01	0.01	<0.01	<0.01	<0.01	<0.01

Note: ^1^ DM: dry matter, CP: crude protein, NDF: neutral detergent fibers, ADF: acid detergent fiber, EE: ether extract, Ca: calcium, P: phosphorus, AA: American Anderson, GE: American Golden Empress, ZM1: Zhongmu No. 1, GN1: Gongnong No. 1; ^2^ Different small letters in the same column indicate significant difference among four alfalfa hay cultivars (*p* < 0.05), while the same letter in the same column indicates no significant difference among four alfalfa hay cultivars (*p* > 0.05).

**Table 4:** Rumen degradable characteristics of CP from various alfalfa hay cultivars %.

Items ^1^	Real-Time Degradability Rate (%)								Degradation Parameters				
	4 h	8 h	12 h	24 h	30 h	36 h	48 h	72 h	a (%)	b (%)	c (%/h)	a + b (%)	ED (%)
AA	46.65 ^ab^	56.70	65.50 ^ab^	74.41 ^a^	77.47 ^a^	79.56 ^a^	80.41 ^a^	81.61 ^a^	31.21	50.20	0.09	81.40 ^a^	68.50 ^a^
GE	48.05 ^a^	57.06	68.64 ^a^	72.05 ^ab^	75.32 ^a^	76.23 ^b^	77.94 ^b^	80.58 ^a^	32.14	46.40	0.11	78.54 ^b^	68.28 ^a^
ZM1	43.45 ^b^	52.84	61.49 ^b^	69.26 ^b^	70.38 ^b^	72.84 ^c^	74.37 ^c^	77.48 ^b^	31.18	44.50	0.08	75.68 ^c^	63.69 ^b^
GN1	45.24 ^ab^	55.92	65.51 ^ab^	72.02 ^ab^	75.56 ^a^	77.84 ^ab^	78.84 ^ab^	81.51 ^a^	30.42	49.95	0.09	80.37 ^a^	67.30 ^b^
SEM	1.071	1.661	1.336	1.552	1.016	0.646	0.602	0.642	3.691	3.482	0.019	0.538	0.552
*p*-value ^2^	0.06	0.301	0.02	0.02	<0.01	<0.01	<0.01	<0.01	0.99	0.60	0.40	<0.01	<0.01

Note: ^1^ a: rapid degradation, b: slow degradation, c: degradable rate constant of slow degradation fraction, ED: effective degradation rate, AA: American Anderson, GE: American Golden Empress, ZM1: Zhongmu No. 1, GN1: Gongnong No. 1; ^2^ Different small letters in the same column indicate significant difference among the four alfalfa hay cultivars (*p* < 0.05), while the same letter in the same column indicates no significant difference among the four alfalfa hay cultivars (*p* > 0.05).

**Table 5:** Rumen degradable characteristics of DM from various alfalfa hay cultivars %.

Items ^1^	Real-time Degradability Rate (%)								Degradation Parameters				
	4 h	8 h	12 h	24 h	30 h	36 h	48 h	72 h	a (%)	b (%)	c (%/h)	a + b (%)	ED (%)
AA	38.16	44.28 ^b^	49.94 ^b^	55.49	61.97 ^b^	64.93 ^b^	66.08 ^bc^	69.31	30.76	40.69	0.05 ^b^	71.45 ^a^	55.27 ^b^
GE	40.32	52.81 ^a^	55.23 ^ab^	63.12	64.85 ^ab^	67.20 ^a^	68.71 ^a^	68.76	28.24	40.16	0.10 ^a^	68.39 ^b^	58.67 ^a^
ZM1	39.52	51.08 ^a^	59.17 ^a^	64.03	66.19 ^a^	67.42 ^a^	68.26 ^ab^	69.87	21.21	47.03	0.12 ^a^	68.24 ^b^	58.90 ^a^
GN1	38.04	43.44 ^b^	49.53 ^b^	56.18	63.74 ^b^	64.17 ^b^	64.61 ^c^	70.17	30.61	40.52	0.05 ^b^	71.12 ^ab^	55.01 ^b^
SEM	1.361	1.221	1.939	2.676	1.055	0.688	0.781	0.732	2.892	2.773	0.012	0.924	0.891
*p*-value ^2^	0.60	<0.01	0.01	0.08	0.03	0.01	0.01	0.55	0.12	0.29	0.01	0.05	0.01

Note: ^1^ a: rapid degradation, b: slow degradation, c: degradable rate constant of slow degradation fraction, ED: effective degradation rate, AA: American Anderson, GE: American Golden Empress, ZM1: Zhongmu No. 1, GN1: Gongnong No. 1. ^2^ Different small letters in the same column indicate significant difference among the four alfalfa hay cultivars (*p* < 0.05), while the same letter in the same column indicates no significant difference among the four alfalfa hay cultivars (*p* > 0.05).

**Table 6:** Rumen degradable characteristics of NDF from various alfalfa hay cultivars %.

Items ^1^	Real-Time Degradability Rate (%)								Degradation Parameters				
	4 h	8 h	12 h	24 h	30 h	36 h	48 h	72 h	a (%)	b (%)	c (%/h)	a + b (%)	ED (%)
AA	11.85 ^b^	21.08 ^b^	21.08 ^a^	34.81 ^bc^	39.52 ^bc^	42.33 ^b^	44.65 ^b^	47.28 ^b^	2.73 ^b^	43.51 ^c^	0.07 ^a^	46.23 ^c^	32.67 ^a^
GE	16.92 ^a^	25.93 ^a^	25.93 ^ab^	40.01 ^a^	41.12 ^ab^	42.59 ^b^	43.65 ^b^	44.43 ^c^	6.89 ^a^	37.94 ^d^	0.08 ^a^	44.83 ^c^	34.03 ^a^
ZM1	10.55 ^b^	15.28 ^c^	15.28 ^c^	32.55 ^c^	37.82 ^c^	39.33 ^c^	44.46 ^b^	47.44 ^b^	2.39 ^b^	47.66 ^b^	0.04 ^b^	50.05 ^b^	30.06 ^b^
GN1	11.22 ^b^	19.92 ^b^	24.93 ^bc^	36.41 ^b^	42.18 ^a^	46.29 ^a^	48.82 ^a^	51.25 ^a^	2.05 ^b^	51.11 ^a^	0.05 ^b^	53.16 ^a^	33.73 ^a^
SEM	0.655	0.873	1.063	0.969	0.787	0.926	1.187	0.741	0.86	0.991	0.001	0.952	0.591
*p*-value ^2^	<0.01	<0.01	<0.01	0.01	0.01	0.02	0.04	<0.01	0.03	<0.01	<0.01	<0.01	0.02

Note: ^1^ a: rapid degradation, b: slow degradation, c: degradable rate constant of slow degradation fraction, ED: effective degradation rate, AA: American Anderson, GE: American Golden Empress, ZM1: Zhongmu No. 1, GN1: Gongnong No. 1. ^2^ Different small letters in the same column indicate significant difference among the four alfalfa hay cultivars (*p* < 0.05), while the same letter in the same column indicates no significant difference among the four alfalfa hay cultivars (*p* > 0.05).

**Table 7:** Rumen degradable characteristics of ADF from various alfalfa hay cultivars %.

Items ^1^	Real-Time Degradability Rate (%)								Degradation Parameters				
	4 h	8 h	12 h	24 h	30 h	36 h	48 h	72 h	a (%)	b (%)	c (%/h)	a + b (%)	ED (%)
AA	8.92 ^b^	13.34 ^c^	21.28 ^bc^	29.64 ^b^	32.81 ^c^	40.45 ^ab^	42.85 ^b^	44.99 ^b^	1.06 ^b^	47.46 ^a^	0.04 ^b^	0.04 ^a^	28.23 ^b^
GE	15.56 ^a^	22.38 ^a^	25.44 ^a^	35.41 ^a^	37.27 ^ab^	38.26 ^bc^	39.55 ^c^	40.89 ^c^	7.28 ^a^	33.89 ^b^	0.07 ^a^	0.07 ^b^	30.73 ^a^
ZM1	8.11 ^b^	12.30 ^c^	19.25 ^c^	29.22 ^b^	35.08 ^bc^	35.69 ^c^	39.85 ^c^	44.78 ^b^	0.48 ^b^	46.66 ^a^	0.04 ^b^	0.04 ^a^	27.01 ^b^
GN1	8.68 ^b^	17.42 ^b^	22.68 ^b^	33.54 ^a^	39.03 ^a^	41.84 ^a^	45.44 ^a^	48.58 ^a^	0.44 ^b^	49.69 ^a^	0.05 ^b^	0.05 ^a^	30.84 ^a^
SEM	0.512	1.054	0.723	0.729	1.018	0.909	0.814	0.923	0.623	1.092	0.003	1.213	0.537
*p*-value ^2^	<0.01	<0.01	<0.01	<0.01	0.05	0.02	0.01	0.01	<0.01	<0.01	<0.01	0.01	<0.01

Note: ^1^ a: rapid degradation, b: slow degradation, c: degradable rate constant of slow degradation fraction, ED: effective degradation rate, AA: American Anderson, GE: American Golden Empress, ZM1: Zhongmu No. 1, GN1: Gongnong No. 1. ^2^ Different small letters in the same column indicate significant difference among the four alfalfa hay cultivars (*p* < 0.05), while the same letter in the same column indicates no significant difference among the four alfalfa hay cultivars (*p* > 0.05).

**Table 8:** Evaluation of feed value from various alfalfa hay cultivars %.

Items ^1^	TDN	DMI	DDM	RFV	RFQ
AA	57.59 ^a^	2.74 ^a^	63.20 ^a^	134.38 ^a^	128.42 ^a^
GE	56.81 ^a^	2.78 ^a^	62.40 ^a^	134.44 ^a^	128.38 ^a^
ZM1	50.59 ^c^	2.24 ^c^	55.95 ^c^	97.07 ^c^	92.06 ^c^
GN1	53.30 ^b^	2.44 ^b^	58.76 ^b^	111.37 ^b^	105.96 ^b^
SEM	0.279	0.026	0.287	0.795	0.778
*p*-value ^2^	<0.01	<0.01	<0.01	<0.01	<0.01

Note: ^1^ TDN: total digestible nutrients, DMI: dry matter intake, DDM: digestible day matter, RFV: relative feed value, RFQ: relative forage quality, and above all are calculated value, AA: American Anderson, GE: American Golden Empress, ZM1: Zhongmu No. 1, GN1: Gongnong No. 1. ^2^ Different small letters in the same column indicate significant difference among the four alfalfa hay cultivars (*p* < 0.05), while the same letter in the same column indicates no significant difference among the four alfalfa hay cultivars (*p* > 0.05).

## Data Availability

The data that support the findings of this study are available from the corresponding author upon reasonable request.

## Abbreviations

AA: American Anderson; GE: American Golden Empress; ZM1: Zhongmu No. 1; GN1: Gongnong No. 1; DM: day matter; CP: crude protein; NDF: neutral detergent fibers; ADF: acid detergent fiber; EE: ether extract; Ca: calcium; P: phosphorus; TDN: total digestible nutrients; DMI: dry matter intake; DDM: digestible dry matter; RFV: relative feed value; RFQ: relative forage quality; a: rapid degradation; b: slow degradation; c: degradable rate constant of slow degradation fraction; ED: effective degradation rate.
